# Assessing the Relationship Between the Type of Internet Use and Internet Addiction in Early and Middle Adolescents: Cross-Sectional Study From Qatar

**DOI:** 10.2196/62955

**Published:** 2025-02-10

**Authors:** Khansa Chemnad, Maryam Aziz, Sanaa Al- Harahsheh, Azza Abdelmoneium, Ahmed Baghdady, Diana Alsayed Hassan, Raian Ali

**Affiliations:** 1 College of Science and Engineering, Hamad Bin Khalifa University Doha Qatar; 2 World Innovation Summit for Health Qatar Foundation Doha Qatar; 3 Doha International Family Institute Qatar Foundation Doha Qatar; 4 World Innovation Summit for Education Qatar Foundation Doha Qatar; 5 Department of Public Health College of Health Sciences QU Health, Qatar University Doha Qatar

**Keywords:** internet addiction, internet use, early adolescence, middle adolescence, mobile phone

## Abstract

**Background:**

With the increasing prevalence of digital technology, adolescent internet addiction (IA) has become a global concern. Excessive internet use, especially among adolescents, has been linked to various negative outcomes such as poor academic performance, social isolation, and mental health issues. Conducted among adolescents of Arab origin, our study addressed the limitations of the literature, which predominantly focuses on Western, educated, industrialized, rich, and democratic populations.

**Objective:**

This study aimed to differentiate between essential and nonessential internet use and how they relate to IA in early and middle adolescents, as well as the relationship between subjective happiness with the amount of time spent on nonessential internet use and IA.

**Methods:**

A cross-sectional survey was conducted among 377 students from 16 schools in Qatar. The survey measured essential and nonessential internet use, subjective happiness with nonessential use, and IA symptoms using the Internet Addiction Diagnostic Questionnaire, as well as participant demographics. To explore age-specific associations, participants were categorized into early (age 11-13 years) and middle (age 14-17 years) adolescents. Factorial analysis, multiple regression, and logistic regression were used for statistical analysis.

**Results:**

Nonessential internet use significantly predicted IA in both early (*P*<.001) and middle (*P*<.001) adolescents, with early adolescents showing a stronger association. Subjective happiness with nonessential internet use negatively predicted IA only in middle adolescents (*P*<.001) as greater dissatisfaction led to a higher IA risk. Essential internet use did not predict IA in either group.

**Conclusions:**

Differentiating between essential and nonessential internet use is crucial in understanding IA. This study highlights the importance of developmental differences in shaping IA symptoms. The findings suggest that interventions aimed at addressing IA should be age specific and focus on addressing nonessential use specifically rather than considering internet use and screen time in general as a single entity. Cultural and regional factors also play a role in shaping internet use patterns and IA in the Middle East, necessitating context-specific, culturally sensitive approaches to IA prevention.

## Introduction

### Background

Adolescent internet addiction (IA) has become a growing concern in today’s society as the use of technology and the internet has become increasingly prevalent in the lives of young people. According to a study conducted by the Pew Research Center, since 2014 to 2015, there has been a modest increase in the proportion of adolescents who said that they used the internet daily or more often [1]. In a follow-up study in 2022, a total of 97% of adolescents said that they used the internet daily compared to 92% of adolescents who said the same in 2014 to 2015. It is understandable that many adolescents struggle with IA given the widespread use of the internet. Studies have shown that approximately 10% of adolescents are at risk of IA, with even higher rates reported in countries such as Hong Kong, China, and South Korea [2-5]. For example, a survey conducted in South Korea found that nearly 30% of junior high school students were either addicted to the internet or at a risk of IA [6]. While some internet use can be beneficial for educational and social purposes, excessive use can lead to negative consequences such as decreased academic performance, social isolation, and even mental health issues [7-9]. Therefore, it is important to understand the prevalence of IA among adolescents and its contributing factors.

IA is defined as the inability to control internet use, leading to disruptions in everyday functioning and symptoms of withdrawal and tolerance [10]. Although not officially recognized as a disorder in the most recent edition of the *Diagnostic and Statistical Manual of Mental Disorders*, IA has received considerable attention in scientific literature and is regarded as a growing concern [11]. Opinions in the literature differ, with some researchers suggesting distinct diagnostic criteria and subtypes of IA, such as video game and online gambling addiction [12]. However, there is still a lack of consensus regarding the internet’s addictive nature and IA diagnostic criteria [13]. While there is still debate regarding the exact nature and criteria for IA, it is clear that excessive internet use can have negative consequences, particularly for adolescents. Understanding the prevalence and factors contributing to IA is important for addressing this growing concern, promoting healthy internet use, and developing appropriate interventions for those who may be affected.

Most psychological research studies predominantly rely on samples from Western, educated, industrialized, rich, and democratic (WEIRD) populations [14]. However, when examining the effects of excessive internet use on adolescent mental health, it is crucial to consider that the experiences of adolescents from non-WEIRD populations may vary owing to many factors, such as different cultural norms and access to technology [15]. This emphasizes the importance of including more diverse samples in research to gain a comprehensive understanding of the global impact of technology on mental health. Cultural context significantly shapes adolescence, with the experiences of young individuals varying widely across different cultures [16]. To address this gap, our study focused on adolescent IA in the Middle East, where limited research on IA has been conducted. In doing so, we aimed to shed light on the specific dynamics of IA in this region and contribute to a more inclusive and comprehensive understanding of this phenomenon.

Qatar has one of the highest internet penetration rates in the world at 99% [17]. The country’s internet users have grown significantly from 69% in 2012 to 100% in 2022 [18]. This increase raises the possibility of an increase in IA prevalence rates, as does the spread of mobile device use and the constant release of new technology updates. Furthermore, adolescents may unintentionally be encouraged to spend more time inside streaming online content, playing online games, and spending time on-screen due to Qatar’s humid and hot weather, which lasts for more than half the year.

In addition to Qatar’s unique technological landscape and climate, broader Middle Eastern cultural factors play a crucial role in shaping adolescent internet use patterns. The region’s complex environment, which is characterized by its unique family dynamics, societal expectations, and cultural values, may contribute to the elevated prevalence of IA among adolescents [19,20]. In various Middle Eastern societies, conservative norms that prioritize modesty and restraint frequently conflict with the liberties that online platforms provide. As adolescents navigate their identities and seek communities that are consistent with their evolving values, this cultural tension can motivate them to engage with the internet differently. In addition, it is also argued that the limited opportunities for social interaction outside the home, along with societal pressures to conform, may encourage adolescents to seek connection and validation online, potentially nurturing addictive behaviors [19]. Culturally informed research can inform tailored intervention strategies that address the specific needs of adolescents in the Middle East. For example, studies suggest that effective interventions should involve families and schools, recognizing the significant role that these environments play in shaping adolescents’ internet behaviors [21,22].

Excessive screen time is often identified as a prominent indicator of IA, contributing to an unhealthy relationship with the internet and development of negative habits [23,24]. In numerous studies examining adolescent IA, the amount of time spent on the web is commonly regarded as a key factor in determining the presence of IA [25,26]. The COVID-19 pandemic has further heightened the reliance on technology for remote learning and socializing among adolescents [27]. The term *screen time* is frequently used without sufficient attention being paid to the specific activities undertaken during that period. However, this lack of specificity has several methodological limitations. To address these limitations, this study aimed to differentiate between the time spent on essential and nonessential internet use activities, providing a more nuanced understanding of IA in adolescents. However, IA is not just a matter of excessive screen time; it has also been linked to mental health issues such as loneliness, low self-esteem, sleep problems, depression, social phobia, and anxiety [28,29]. Adolescents who struggle with these conditions may turn to the internet as a coping mechanism, further exacerbating their addiction [30]. Ultimately, IA can have a significant negative impact on adolescents’ lives, from decreased academic performance to strained relationships with loved ones [31].

Adolescence is a crucial period for identity exploration and formation during which individuals may encounter situations in which their emerging beliefs and values conflict with societal expectations or their own behaviors, which may lead to cognitive dissonance [32]. This aspect of adolescent development is relevant to the exploration of subjective feelings regarding digital technology use and its association with IA. Cognitive dissonance theory offers an understanding of this relationship by examining potential conflicts arising from adolescents’ subjective feelings regarding their digital technology use and addictive behavior. According to the cognitive dissonance theory, individuals experience psychological discomfort when they hold contradictory beliefs, attitudes, or behaviors [33]. In the context of digital technology use and IA, cognitive dissonance may arise when adolescents recognize the negative consequences of excessive digital technology use but continue to engage in it. The literature suggests that subjective positive feelings about one’s own life conditions are negatively correlated with IA [34]. It is reasonable to hypothesize that similar correlations may exist between subjective feelings regarding digital technology use and IA. However, there is a lack of specific studies exploring this association. Given this gap in the literature, this study aimed to address this by examining the association between subjective feelings about digital technology use and IA.

Adolescents are growing up in an era characterized by rapid technological advancements, and the use of smartphones has become a common part of their daily lives. This pervasive technology has greatly influenced their interactions with the internet, particularly social networking sites [35]. Consequently, this has had a profound impact on various aspects of their lives, including social dynamics, cognitive development, and emotional well-being. The literature indicates notable distinctions between early and middle adolescents in terms of cognitive and psychological development [36]. It is critical to distinguish between early and middle adolescence when investigating adolescent IA owing to the distinct developmental traits, experiences, and coping strategies that arise throughout these separate phases of adolescence [37]. Early adolescence is a critical period defined by the start of puberty and the transition from childhood to adolescence. This stage is distinguished by rapid physical, cognitive, and social changes, as well as identity exploration and self-concept construction [38,39]. Furthermore, early adolescence is a critical time for the initiation and development of mental health issues [40]. Middle adolescence is characterized by managing the complications of moving into young adulthood [41]. Individuals become more self-sufficient, engage in complex social connections, and gain feelings of autonomy [42]. If these aspects of development are not addressed properly during adolescence, consequences may have a lasting impact in their adulthood. Researchers must examine the diverse vulnerabilities, risk factors, and patterns of internet use among early and middle adolescents while studying IA during these different phases. Early adolescents may be more vulnerable to IA because they are constantly trying new things, including online platforms, to define their identities and connect with friends [39]. Middle adolescence, on the other hand, is characterized by the emergence of more stable patterns of behavior, including internet use, as adolescents approach young adulthood and deal with increasing academic and social demands [43]. Furthermore, research has shown that the characteristics that influence IA change between early and middle adolescence [21]. For example, parental supervision and advice may have a greater effect among early adolescents, but peer influence and social support may become more significant in middle adolescence [44,45]. Furthermore, in middle adolescence, the onset of academic commitment and the need for self-regulation might alter internet use patterns and addiction risks. IA has become a growing concern among adolescents, with symptoms presenting both in relation to oneself and in interactions with others. However, the cognitive development and behavioral patterns of early and middle adolescents differ significantly, suggesting the need for a critical examination of IA symptoms according to age. Although previous research has highlighted the multifaceted nature of IA symptoms, little attention has been paid to the potential differential manifestation of symptoms based on the age of adolescents. Understanding how IA symptoms manifest differently in early and middle adolescence is crucial for tailoring prevention and intervention efforts. Understanding the differences between early and middle adolescence is critical when examining IA. Our study aimed to address the existing knowledge gap by investigating aspects that have not been thoroughly explored in the field of adolescent IA.

Adolescent technology use can be categorized into distinct types depending on type of use: essential and nonessential [46,47]. Activities that are essential for everyday life, such as educational pursuits or personal improvement–related activities, are included in essential use. On the other hand, nonessential use includes leisure activities such as gaming, social media engagement for nonspecific developmental purpose, and other forms of entertainment [46]. Although researchers have investigated the correlations between excessive internet use and IA, there is a noticeable absence of studies that explicitly distinguish between essential and nonessential use patterns. We suggest that the use of the internet for essential activities related to education and personal development can improve efficiency and time management and may not be linked to IA. In contrast, the excessive use of the internet for nonessential activities may help change mood and increase bonding but may also result in diminished productivity and potential adverse consequences [48]. Given this distinction, our study endeavored to examine the distinct effects of essential versus nonessential internet use on adolescents, thereby contributing to resolving a critical gap in the current literature and offering valuable insights for comprehending healthy internet use patterns.

### Objectives

While extensive research has been conducted on IA among adolescents, several significant gaps remain. First, there is a lack of studies focusing on non-WEIRD populations, particularly in the Middle East, where unique cultural factors may influence IA. Second, the potential significance of the distinction between essential and nonessential internet use in relation to IA has not been adequately investigated. Third, there is a scarcity of research on the correlation between subjective emotions related to the use of digital technology and IA. Finally, while the developmental differences between early and middle adolescence are well documented, there are few studies that have investigated the potential differences in the manifestation of IA symptoms and their relationship to technology use across these age groups. We aimed to answer the following three research questions (RQs) through this study:

1. Do essential and nonessential types of internet use predict IA in early and middle adolescents? (RQ 1)
2. Can subjective happiness with nonessential internet use predict adolescent IA? (RQ 2)
3. Does the impact of time spent on essential and nonessential internet use, as well as subjective feelings regarding nonessential internet use, on IA differ between the early and middle adolescence stages? (RQ 3)

## Methods

### Participants

This study involved school students residing in Qatar, and their participation was secured through web-based surveys administered via SurveyMonkey (SurveyMonkey Inc). The survey invitation was sent out to multiple schools in Qatar through an open call using the mailing list of head teachers and educators. One of the authors works at an education policy institute with a network of schools that attend events and participate in research activities. This facilitated outreach. A total of 16 schools accepted the study invitation, comprising an equal number of public (n=8, 50%) and private (n=8, 50%) schools. Public schools in Qatar are government funded and managed by the government and primarily enroll Qatari citizens as they provide free education with Arabic as the language of instruction. These schools are also gender segregated, meaning that male and female students attend separate institutions. Private schools, on the other hand, are funded by tuition fees and managed by private organizations and predominantly enroll expatriate students. These schools follow a variety of government-reviewed international curricula and are typically not gender segregated. Data were collected between March 2022 and May 2022.

Before administering the survey, students were provided with an induction session by their head teacher, who explained the distinction between essential and nonessential internet use. Essential use was defined as internet activities connected to study, academic work, and personal development, whereas nonessential use was defined as activities that do not directly contribute to these aims. This briefing was designed to establish a common understanding among participants, guaranteeing that their answers precisely reflected these definitions. The authors acknowledge the difficulty in standardizing what adolescents consider essential versus nonessential. Therefore, our study and results should be interpreted as reflecting the subjective views and self-reports of the adolescent participants. This interpretation follows an induction on general understanding, which aimed to reduce diversity in perceptions.

We used the formula by Green [49] to calculate the optimal sample size for our research. The formula indicates that a linear regression analysis requires a minimum sample size of 50 plus 8 times the number of independent variables *p* (50 + 8 × *p*). This indicates that a minimum sample size of 74 participants was suitable for our study. In addition, to ensure correlation stability [50], our aim was to have a sample size of >250 individuals. This sample size is consistent with recommendations for maintaining statistical power in studies with multiple predictors [49,50]. Furthermore, the male sample was deemed too small to segregate the analysis by gender.

After excluding participants who did not meet the inclusion criteria, such as those who provided incomplete responses, 64.3% (377/586) of the initial students were included in the analysis, which fulfilled the criteria of a 5% margin of error at the 95% CI. Of these 377 students, 86 (22.8%) were male and 291 (77.2%) were female. The gender distribution in our sample reflects the timing of data collection, which coincided with final exams, as well as the demographic characteristics of the responding schools. Specifically, 12% (2/16) of the schools that responded to the survey were predominantly female-only public schools, which significantly contributed to the observed gender imbalance. It is important to note that gender was not included as a variable in our analysis as the study’s primary focus was on essential and nonessential internet use. On the basis of the estimates of the sample size and the need for adequate statistical power in our regression analysis, it was not possible to divide the sample by gender for separate analyses.

The average age of the participating adolescents was 13.19 (SD 1.24; range 11-17) years. The participants in the study were requested to explicitly provide their ages. Participants were categorized into 2 groups: early adolescents (aged 11-13 years) and middle adolescents (aged 14-17 years). The classification of adolescents into these developmental phases was determined based on the existing literature [51].

### Ethical Considerations

This study was approved by the institutional review board of Hamad Bin Khalifa University (QBRI-IRB 2021-05-094). School permissions were obtained to distribute the survey. The parents and adolescents were informed beforehand of the study, and their written informed consent and assent were collected. Participants were informed about the study’s purpose, and their involvement was voluntary, allowing them to skip questions or withdraw from the survey if desired. During data collection, no identifiable information was collected. The researchers ensured that all deidentified data were stored on a password-protected laptop that only the primary investigators of the study had access to. Participants were not compensated for taking part in this study.

### Measures

#### Overview

The survey included questions regarding participants’ demographics, digital technology use, subjective happiness with nonessential internet use, and IA. The questionnaire was distributed in both English and Arabic to accommodate language preferences, and the accuracy of the Arabic translation was ensured using the back translation method [52] (see Multimedia Appendix 1 for both versions of the survey). Our study included a comprehensive and multistep procedure for validating the survey questions to establish its reliability and suitability for the adolescent population. We began by conducting think-aloud protocols with a subset of 3 adolescents, asking them to verbalize their thought processes while answering the survey questions [53]. This approach helped us identify potential ambiguities or misunderstandings, leading to revisions in translation that ensured clarity and suitability for the target age group yet without departing from the original meaning of the questions in the English version. In total, 3 authors with extensive expertise dealing with adolescent groups in the Arab area offered expert advice. Furthermore, the questions in the survey were culturally appropriate, did not use any jargon, and were free of unclear language.

#### Internet Addiction Diagnostic Questionnaire

The Internet Addiction Diagnostic Questionnaire (IADQ) consists of 8 items, each representing a symptom used to identify IA [10]. The questionnaire uses a binary response format, with participants indicating *no* or *yes* to each symptom. The total score on the IADQ ranges from 0 to 8 and is obtained by summing the values assigned to each of the 8 binary questions. The symptoms assessed by the IADQ are *preoccupation* (question 1), *tolerance* (question 2), *unsuccessful efforts to limit or stop Internet usage* (question 3), *withdrawal* (question 4), *loss of control of time spent on the Internet* (question 5), *risk/lose relationships or opportunities* (question 6), *lies to conceal the extent of involvement* (question 7), and *dysfunctional coping* (question 8) [54]. Participants who answered *yes* to ≥5 symptoms were categorized as addicted to the internet (dependent internet users), whereas the others were categorized as nondependent internet users. Previous studies on the IADQ have reported Cronbach α values ranging from 0.60 to 0.72 [55]. The reliability of the IADQ in this study was deemed acceptable, with a Cronbach α value of 0.66 [56] and a McDonald ω value of 0.67. In addition, the research conducted by Lozano-Blasco et al [57] found that using lesser answer categories led to decreased Cronbach α values. The dichotomous character of the questions in the IADQ, which means that they only allow for replies of *yes* or *no*, leads to lower Cronbach α values that are within the acceptable range for dependability. To establish content validity [58], the initial item pool was reviewed by a panel of 3 experts who has extensive experience working with adolescents in the Arab region. This process resulted a questionnaire that comprehensively covered the construct of interest.

#### Essential and Nonessential Internet Use

Adolescents were presented with a set of questions regarding their use of digital technology for both essential and nonessential purposes on both weekends and weekdays. The weekend is officially observed on Fridays and Saturdays in Qatar. By focusing on weekdays and weekends separately, we aimed to capture any behavioral variations influenced by the shift from weekend to workweek and vice versa. Participants were asked the following questions to encompass four different situations: (1) How many hours do you use digital technology for study purposes daily on weekdays (Sunday-Thursday)? (2) How many hours do you use digital technology for nonessential reasons daily on weekdays (Sunday-Thursday)? (3) How many hours do you use digital technology for study purposes daily on weekends (Friday and Saturday)? (4) How many hours do you use digital technology for nonessential reasons daily on weekends (Friday and Saturday)?

Essential internet use on weekdays and weekends was combined, and their average was used to compute essential internet use time. Nonessential internet use on weekdays and weekends was combined, and their average was taken to compute nonessential internet use time.

#### Subjective Happiness With Nonessential Internet Use Time

Adolescents were asked to express their feelings regarding the extent of their nonessential use of digital technology. Their responses were collected using a five-point Likert scale: (1) “I am happy with it,” (2) “I am somewhat happy with it,” (3) “Neither happy nor unhappy with it,” (4) “I am somewhat unhappy with it,” and (5) “I am unhappy with it.”

### Statistical Analysis

Multiple linear regression analyses were conducted separately for early and middle adolescents to examine the relationship between nonessential internet use, essential internet use, and subjective happiness with nonessential internet use time and their IA status. The significance level for the statistical tests was set at *P*<.05. All statistical analyses were carried out using JASP (version 0.17.1) [59].

Exploratory factor analysis with oblique (promax) rotation was performed [60]. The determination of the number of significant components to retain for rotation was based on two criteria: (1) the scree plot indicating the number of extracted factors and (2) ensuring that the factor solution allowed for a coherent interpretation of the results.

## Results

### Descriptive Statistics

The descriptive statistics of the participants are presented in Table 1.

**Table 1:** Descriptive statistics—internet use patterns and internet addiction (IA).

Variable				Early adolescents (n=242)		Middle adolescents (n=135)
Essential internet use time (h), mean (SD)				2.22 (1.76)		2.96 (2.12)
Nonessential internet use time (h), mean (SD)				4.45 (2.74)		4.52 (2.35)
**Subjective happiness with nonessential internet use time, n (%)**						
	“I am happy with it” (rating of 1)			90 (37.2)		44 (32.6)
	“I am somewhat happy with it” (rating of 2)			77 (31.8)		40 (29.6)
	“Neither happy nor unhappy with it” (rating of 3)			46 (19)		35 (25.9)
	“I am somewhat unhappy with it” (rating of 4)			17 (7)		11 (8.1)
	“I am unhappy with it” (rating of 5)			12 (5)		5 (3.7)
**IA**						
	Total IA score (0-8), mean (SD)			3.26 (1.98)		3.47 (2.17)
	**IA prevalence, n (%)**					
		Addicted internet users	63 (26)		50 (37)	
		Nonaddicted internet users	179 (74)		85 (63)	

### Exploratory Factor Analysis of IA Symptoms

To identify underlying dimensions within the IA symptoms and reduce the data into meaningful factor scores, an exploratory factor analysis was conducted on the dataset. The Kaiser-Meyer-Olkin (KMO) test, a measure of sampling adequacy, was used in this study to evaluate multicollinearity in the data to determine the feasibility of conducting a factor analysis. The overall KMO measure was 0.76, denoting “middling” adequacy. Individual KMO measures were all of >0.68, ranging from the “mediocre” to the “marvelous” categories according to the classification system by Kaiser and Rice [61]. The Bartlett test of sphericity was statistically significant (*P*<.001), indicating that the data were likely factorizable.

The analysis extracted 2 components, which accounted for 26% of the variance. Our scree plot was compatible with the 2-factor model. We interpreted the 2 factors as representing internal IA symptoms (factor 1; 18% of the total variance)—those symptoms primarily related to the individual’s self, such as preoccupation with internet use—and external IA symptoms (factor 2; 9% of the total variance)—those symptoms primarily related to interaction with others. Factor loadings for factor 1 and factor 2 ranged from 0.31 to 0.55 and from 0.33 to 0.78, respectively. Factor scores were derived by adding the scores of individual items within each empirical domain (sum score) and dividing the sum scores by the total number of items (mean item score). The component loadings and uniqueness of the rotated solutions are presented in Table 2.

**Table 2:** Factor analysis of internet addiction symptoms.

Item	Factor 1 loadings	Factor 2 loadings	Uniqueness
“Preoccupation” (question 1)	0.49	—^a^	0.79
“Tolerance” (question 2)	0.31	—	0.91
“Unsuccessful efforts to limit or stop Internet usage” (question 3)	0.50	—	0.76
“Withdrawal” (question 4)	0.55	—	0.62
“Loss of control of time spent on the Internet” (question 5)	0.50	—	0.77
“Risk/lose relationships or opportunities” (question 6)	—	0.78	0.48
“Lies to conceal the extent of involvement” (question 7)	—	0.33	0.75
“Dysfunctional coping” (question 8)	0.45	—	0.79

^a^The factor does not comprise this item.

### Early Adolescents

#### Predictors of Total IA Scores

##### Overview

Multiple regression analysis was used to determine factors that predicted early adolescents’ total IA scores and internal IA symptoms. No outliers were observed in the data. The Pearson correlation was also used to analyze the associations between total IA, which was the dependent variable, and the independent variables of subjective happiness with nonessential internet use time, essential internet use time, and nonessential internet use time.

##### Correlation Analysis of Variables

The correlations between the variables are presented in Table 3.

**Table 3:** Pearson correlation table between the variables in early adolescents.

	Total IA^a^	Internal IA symptoms	External IA symptoms	Subjective happiness with nonessential internet use time	Essential internet use time	Nonessential internet use time
Total IA	1	—^b^	—	—	—	—
Internal IA symptoms	0.95^c^	1	—	—	—	—
External IA symptoms	0.58^c^	0.30^c^	1	—	—	—
Subjective happiness with nonessential internet use time	0.04	0.03	0.03	1	—	—
Essential internet use time	0.07	0.04	0.10	0.04	1	—
Nonessential internet use time	0.41^c^	0.41^c^	0.18^d^	0.07	0.12	1

^a^IA: internet addiction.

^b^Not applicable.

^c^*P*<.001.

^d^*P*<.01.

##### Regression Analysis of Total IA Scores

Table 4 shows the results of the multiple regression that was run to predict the total IA score from the subjective happiness with nonessential internet use time, essential internet use time, and nonessential internet use time among early adolescents. The linearity of the sample was assessed by examining the residuals versus predicted plots, whereas residuals versus dependent plots were used to evaluate homoscedasticity in the data. All the assumptions of linearity, normality, homoscedasticity, and multicollinearity were satisfied. Residuals were independent, as assessed via a Durbin-Watson statistic of 1.93. There was no evidence of multicollinearity, as assessed via tolerance values of >0.1. The assumption of normality was met, as assessed using a *Q*-*Q* plot. The multiple regression model significantly predicted the total IA score (*F*_3, 238_=15.72; *P*<.001; *R*^2^=0.17; adjusted *R*^2^=0.15). Within the model, nonessential internet use time (β=.40; *P*<.001) was the only significant predictor of early adolescents’ total IA scores.

**Table 4:** Multiple linear regression analysis predicting internet addiction in early adolescents^a^.

Predictor	Standardized ꞵ	*t* test (*df*)	*P* value
Constant	—^b^	5.73 (3)	<.001
Subjective happiness with nonessential internet use time	.00923	0.16 (3)	.88
Essential internet use time	.02	0.36 (3)	.72
Nonessential internet use time	.40	6.74 (3)	<.001

^a^*R*^2^=0.17; adjusted *R*^2^=0.15; *F*_3, 238_=15.72.

^b^Not applicable.

#### Predictors of Internal and External IA Symptoms

Multiple regression analysis was used to determine factors that predicted internal IA symptoms with the independent variables of subjective happiness with nonessential internet use time, essential internet use time, and nonessential internet use time, and an ordinal logistic regression model was used to predict external IA symptoms. Table 5 shows the results of the multiple regression that was run to predict the internal IA symptom score from the subjective happiness with nonessential internet use time, essential internet use time, and nonessential internet use time. The linearity of the sample was assessed by examining the residuals versus predicted plots, whereas residuals versus dependent plots were used to evaluate homoscedasticity in the data. All the assumptions of linearity, normality, homoscedasticity, and multicollinearity were satisfied. Residuals were independent, as assessed via a Durbin-Watson statistic of 1.98. There was no evidence of multicollinearity, as assessed via tolerance values of >0.1. The assumption of normality was met, as assessed using a *Q*-*Q* plot. The multiple regression model significantly predicted the internal IA symptom score (*F*_3, 238_=15.95; *P*<.001; *R*^2^=0.17; adjusted *R*^2^=0.16). Within the model, nonessential internet use time (β=.41; *P*<.001) was the only significant predictor of early adolescents’ internal IA symptoms.

**Table 5:** Multiple regression analysis predicting internal internet addiction symptoms in early adolescents^a^.

Predictor	Standardized ꞵ	*t* test (*df*)	*P* value
Constant	—^b^	6.18 (3)	<.001
Subjective happiness with nonessential internet use time	.00318	0.05 (3)	.96
Essential internet use time	−.00431	−0.07 (3)	.94
Nonessential internet use time	.41	6.86 (3)	<.001

^a^*R*^2^=0.17; adjusted *R*^2^=0.16; *F*_3, 238_=15.95.

^b^Not applicable.

An ordinal logistic regression analysis was conducted to examine the relationship between the 3 predictor variables (ie, subjective happiness with nonessential internet use time, essential internet use time, and nonessential internet use time) and the outcome variable (external IA symptoms; Table 6). The variables included in the ordinal logistic regression analysis met the assumptions of the statistical model—the dependent variable (ie, external IA symptoms) was treated as an ordinal variable, and the independent variables (ie, subjective happiness with nonessential internet use time, essential internet use time, and nonessential internet use time) were treated as continuous variables. The assumption of no multicollinearity was assessed by examining the variance inflation factors (VIFs), and the result (VIFs<1.1) indicated no multicollinearity. The results revealed that nonessential internet use time was associated with increased external IA symptoms (odds ratio [OR] 1.13, 95% CI 1.02-1.24; *P*=.02). Meanwhile, essential internet use time was not associated with any change in external IA symptoms (OR 1.11, 95% CI 0.96-1.28; *P*=.17). Similarly, subjective happiness with nonessential internet use time was not associated with any change in external IA symptoms (OR 0.99, 95% CI 0.78-1.25; *P*=.94) in early adolescents.

**Table 6:** Ordinal logistic regression predicting external internet addiction symptoms in early adolescents.

Predictor	OR^a^ (95% CI; SE)	*P* value
Subjective happiness with nonessential internet use time	0.99 (0.78-1.25; −0.01)	.94
Essential internet use time	1.11 (0.96-1.28; 0.10)	.17
Nonessential internet use time	1.13 (1.02-1.24; 0.12)	.02

^a^OR: odds ratio.

Binary logistic regression was conducted to examine the relationship between subjective happiness with nonessential internet use time and the likelihood of being a dependent internet user (addicted) compared to a nondependent internet user (unaddicted) in early adolescents. The reference category, representing nondependent internet users, was coded as class 1. The OR of 0.93 (95% CI 0.73-1.20) indicated the effect size. However, the results did not reveal a significant association between subjective happiness with nonessential internet use time and IA (*P*=.58).

### Middle Adolescents

#### Predictors of Total IA Scores

##### Overview

Multiple regression analysis was used to determine the factors that predicted middle adolescents’ total IA scores and internal IA symptoms. No outliers were observed in the data. The Pearson correlation was also used to analyze the associations between total IA, which was the dependent variable, and the independent variables of subjective happiness with nonessential internet use time, essential internet use time, and nonessential internet use time.

##### Correlation Analysis of Variables

The correlations between the variables are presented in Table 7.

**Table 7:** Pearson correlation table between the variables—middle adolescents.

	Total IA^a^	Internal IA symptoms	External IA symptoms	Subjective happiness with nonessential internet use time	Essential internet use time	Nonessential internet use time
Total IA	1	—^b^	—	—	—	—
Internal IA symptoms	0.95^c^	1	—	—	—	—
External IA symptoms	0.66^c^	0.39^c^	1	—	—	—
Subjective happiness with nonessential internet use time	0.34^c^	0.28^d^	0.32^c^	1	—	—
Essential internet use time	0.21^e^	0.15	0.26^d^	0.18^e^	1	—
Nonessential internet use time	0.35^c^	0.33^c^	0.24^d^	0.13	0.05	1

^a^IA: internet addiction.

^b^Not applicable.

^c^*P*<.001.

^d^*P*<.01.

^e^*P*<.05.

##### Regression Analysis of Total IA Scores

Table 8 shows the results of the multiple regression that was run to predict middle adolescents’ total IA score from the subjective happiness with nonessential internet use time, essential internet use time, and nonessential internet use time. All the assumptions of linearity, normality, homoscedasticity, and multicollinearity were satisfied. The linearity of the sample was assessed by examining the residuals versus predicted plots, whereas residuals versus dependent plots were used to evaluate homoscedasticity in the data. Residuals were independent, as assessed via a Durbin-Watson statistic of 1.83. There was no evidence of multicollinearity, as assessed via tolerance values of >0.1. The assumption of normality was met, as assessed using a *Q*-*Q* plot. The multiple regression model significantly predicted the total IA score (*F*_3, 131_=12.96; *P*<.001; *R*^2^=0.23; adjusted *R*^2^=0.21). Within the model, nonessential internet use time (β=.31; *P*<.001) and subjective happiness with nonessential internet use time (β=.27; *P*<.001) were significant predictors of middle adolescents’ total IA score.

**Table 8:** Multiple linear regression analysis predicting internet addiction in middle adolescents^a^.

Predictor	Standardized ꞵ	*t* test (*df*)	*P* value
Constant	—^b^	1.16 (3)	.25
Subjective happiness with nonessential internet use time	.27	3.44 (3)	<.001
Essential internet use time	.15	1.90 (3)	.06
Nonessential internet use time	.31	3.94 (3)	<.001

^a^*R*^2^=0.23; adjusted *R*^2^=0.21; *F*_3, 131_=12.96.

^b^Not applicable.

#### Predictors of Internal and External IA Symptoms

Multiple regression analysis was used to determine factors that predicted internal IA symptoms with the independent variables of subjective happiness with nonessential internet use time, essential internet use time, and nonessential internet use time, and ordinal logistic regression was used to predict external IA symptoms. Table 9 presents the results of the multiple regression. The linearity of the sample was assessed by examining the residuals versus predicted plots, whereas residuals versus dependent plots were used to evaluate homoscedasticity in the data. All the assumptions of linearity, normality, homoscedasticity, and multicollinearity were satisfied. Residuals were independent, as assessed via a Durbin-Watson statistic of 1.82. There was no evidence of multicollinearity, as assessed via tolerance values of >0.1. The assumption of normality was met, as assessed using a *Q*-*Q* plot. The multiple regression model significantly predicted the internal IA symptom score (*F*_3, 131_=9.15; *P*<.001; *R*^2^=0.17; adjusted *R*^2^=0.15). Within the model, nonessential internet use time (β=.29; *P*<.001) and subjective happiness with nonessential internet use time (β=.22; *P*=.007) were significant predictors of middle adolescents’ internal IA symptoms.

**Table 9:** Multiple regression analysis predicting internal internet addiction symptoms in middle adolescents^a^.

Predictor	Standardized ꞵ	*t* test (*df*)	*P* value
Constant	—^b^	2.10 (3)	.04
Subjective happiness with nonessential internet use time	.22	2.76 (3)	.007
Essential internet use time	.10	1.20 (3)	.23
Nonessential internet use time	.29	3.65 (3)	<.001

^a^*R*^2^=0.17; adjusted *R*^2^=0.15; *F*_3, 238_=15.95.

^b^Not applicable.

An ordinal logistic regression analysis was conducted to examine the relationship between the 3 predictor variables (ie, subjective happiness with nonessential internet use time, essential internet use time, and nonessential internet use time) and the outcome variable (external IA symptoms; Table 10). The variables included in the ordinal logistic regression analysis met the assumptions of the statistical model—the dependent variable (ie, external IA symptoms) was treated as an ordinal variable, and the independent variables (ie, subjective happiness with nonessential internet use time, essential internet use time, and nonessential internet use time) were treated as continuous variables. The assumption of no multicollinearity was assessed by examining the VIFs, and the result (VIFs<1.1) indicated no multicollinearity. The results revealed that all 3 predictors were associated with changes in the external IA symptoms. Nonessential internet use time was associated with an increase in external IA symptoms (OR 1.19, 95% CI 1.03-1.39; *P*=.02). Essential internet use time was associated with an increase in external IA symptoms (OR 1.24, 95% CI 1.05-1.46; *P*=.01). Similarly, a higher unhappiness with nonessential internet use time was associated with increased external IA symptoms (OR 1.75, 95% CI 1.28-2.44; *P*<.001).

**Table 10:** Ordinal logistic regression predicting external internet addiction symptoms in middle adolescents.

Predictor	OR^a^ (95% CI; SE)	*P* value
Subjective happiness with nonessential internet use time	1.75 (1.28-2.44; 0.56)	<.001
Essential internet use time	1.24 (1.05-1.46; 0.21)	.02
Nonessential internet use time	1.19 (1.03-1.39; 0.18)	.01

^a^OR: odds ratio.

A binary logistic regression was performed to assess the impact of subjective happiness with nonessential internet use time on the likelihood of being a dependent internet user (addicted) versus a nondependent internet user (unaddicted). The reference category was coded as class 1, representing nondependent internet users. The OR of 0.66 (95% CI 0.47-0.92) indicated the effect size. The *F*-measure was 75%. The results indicated a significant association between subjective happiness with nonessential internet use time and IA (*P*=.01), suggesting that higher levels of unhappiness were associated with a decreased likelihood of being unaddicted to the internet.

## Discussion

### Principal Findings

This study aimed to enhance our understanding of adolescent IA by addressing 3 key RQs. First, we examined the significance of differentiating between essential and nonessential internet use in relation to IA, offering a more nuanced view of IA beyond that of total screen time that is found in previous research. Second, we investigated whether subjective happiness linked to time spent on nonessential internet use could predict IA in adolescents. Finally, by studying early and middle adolescents separately, we identified age-specific differences in how time spent on essential and nonessential internet use and the related happiness contribute to IA.

Consistent with the extensive existing literature on IA, our study demonstrated that excessive engagement in nonessential internet activities, such as social media and online gaming, positively correlate with IA overall [62-64]. Furthermore, in our study, essential internet use did not exhibit a significant correlation with IA (*P*=.94 for early adolescents and *P*=.06 for middle adolescents), suggesting that essential internet activities such as remote learning or using the internet for school-required work may not carry the same level of addiction risk. This is similar to the results obtained in other studies [65,66]; Salubi et al [65] found that there was no correlation between essential internet use and IA among 390 surveyed university students in South Africa, whereas Pjevac et al [66] found that IA was lowest among adolescents who mostly used the internet for school purposes. This distinction of the purposes of internet use enables the development of targeted interventions and strategies to effectively address this issue. As digital technology becomes increasingly integral to tasks that once operated without it, acknowledging this technological shift is essential in our evolving digital society. It is necessary to recognize the evolving landscape of digital technology use and interpret digital technology use with caution, as emphasized by Squire and Steinkuehler [67], before delving into the complexities of IA. This caution entails considering the specific activities carried out on the internet, the motivations driving them, and the duration for which they occur.

Our study highlights similarities and key differences in IA between early and middle adolescents as our results revealed that, among early adolescents, nonessential internet use significantly predicted IA and its internal symptoms (related to the self). However, middle adolescents exhibited a more complex relationship with IA as both nonessential and essential internet use, in addition to unhappiness with nonessential internet activities, significantly predicted IA and its external symptoms (related to others).

Our results suggest that early adolescents are more likely to develop IA and experience internal IA symptoms as a result of nonessential internet use, whereas this influence seems to decrease as they get older. This finding is in line with the results of a meta-analysis (of 20 studies) on IA in adolescents reporting that IA is inversely proportional to age [57]. This difference suggests that the impact of nonessential digital technology use on IA may vary according to the developmental stage of adolescents. One possible explanation is that early adolescents are actively navigating their new identity and seeking peer acceptance, which can make them more susceptible to excessive use of digital technology [68]. The pressure to conform to peers’ behaviors and expectations may override their ability to exercise self-control and resist the allure of digital technology [69]. Furthermore, in early adolescents, only nonessential internet use increased the likelihood of external IA symptoms. Our results are similar to those of a recent study that examined sociodemographic factors related to IA among a sample of 1664 adolescents in Serbia. The study reported the lowest IA rates among the younger adolescents and among those who used the internet for the purpose of schoolwork [66]. Our results could be interpreted as early adolescents not facing the same academic pressures and responsibilities that drive older adolescents to the excessive use of the internet for essential activities. Instead, early adolescent IA is more related to recreational online activities, underscoring the importance of tailoring preventive interventions to focus on nonessential use, such as social media and gaming, to equip early adolescents to develop healthier digital habits. Parental involvement and supervision play a vital role in preventing IA in early adolescents. Actively engaging with children, establishing open communication channels, and monitoring their digital technology use can create a supportive environment and set boundaries for healthy digital behaviors [27,70]. Emphasizing balance is also essential, highlighting the need for a well-rounded lifestyle that includes academic tasks, physical activities, face-to-face interactions, and nondigital recreational pursuits [71]. Self-control in early adolescents may be enhanced through self-control–training activities and participation in self-control–promoting programs [72].

In contrast to the results for early adolescents, our results demonstrated a more complex relationship between internet use and IA in middle adolescents. Our study found that, while nonessential internet use remains a significant predictor of IA, subjective happiness with nonessential internet activities also plays a role. Specifically, the unhappier middle adolescents were with the time spent on nonessential internet use, the more likely they were to experience IA. These findings are consistent with the findings of a similar study that surveyed a sample of adolescents and young adults (aged 15-24 years) and adults (aged 25-64 years), where dissatisfaction with smartphone use was linked to higher problematic internet use [73]. Our results contribute to the literature as there is a lack of research examining subjective happiness with digital technology use in relation to IA in adolescents.

This can be attributed to the increased academic pressure and the need to manage uncertainty, which drive middle adolescents to spend more time on essential internet activities, such as online educational tasks. The combination of information overload, academic demands, and the desire to avoid having to deal with uncertainty may lead to excessive internet use, further exacerbating IA.

Moreover, middle adolescents exhibited a broader range of factors influencing their external IA symptoms.

While, in early adolescents, only nonessential internet use increased the likelihood of external IA symptoms, in middle adolescents, both nonessential and essential internet use, as well as unhappiness with the time spent on nonessential internet use, increased external IA symptoms. This can be attributed to the intensified academic pressure in combination with information overload, leading them to neglect other aspects of their lives and driving them to the excessive use of the internet to meet academic demands [74,75]. The combination of these factors heightens the risk of IA [76-78]. Furthermore, these academic pressures can further impact social interactions as middle adolescents prioritize academic performance, leading them to show signs of stress in their engagement with others [74,79], manifesting in external symptoms of IA, as observed in our study. Recognizing the unique challenges faced by adolescents at different developmental stages is crucial for effectively tailoring preventive measures and interventions.

While our study focused on examining individual factors related to IA, considering factors related to the specific context in which our results were obtained should not be overlooked and could provide a more holistic understanding of IA among adolescents. Adolescents’ internet use and their susceptibility to IA are influenced by cultural values, societal norms, and regional habits. For instance, in the Middle East, where our study was conducted, cultural expectations regarding academic achievement and family roles may contribute to heightened stress and pressure on adolescents [80,81], leading them to spend more time on online activities such as social media and gaming. The emphasis on academic success in our context combined with limited outdoor recreational opportunities due to the extremely hot climate might be driving adolescents to spend more time indoors and engage in online recreational activities, increasing their risk of IA.

In addition, the rapid digital transformation in the Middle East has created new challenges and opportunities for adolescents [82,83]. The increased availability of smartphones and internet access as well as a growing digital culture have made it easier for people to spend long hours on the web. While there are positive aspects of digitalization, it also raises concerns about the potential for excessive use and the development of IA. The addition of environmental factors such as the local affluent culture of convenience, the wide availability of digital devices even at very young ages, and the wide presence of high-speed internet further influences how adolescents interact with digital technology.

Our study has important implications for the field of IA and for promoting healthier digital technology use habits in adolescents. The findings suggest that interventions aimed at addressing IA should focus on nonessential use specifically, particularly in early adolescents, rather than considering internet use as a whole. Because IA is a complex outcome influenced by multiple physical, psychological, and technological factors rather than a single issue, this targeted approach can help effectively address the factors contributing to IA while considering the developmental factors of adolescents. While there is a dearth of data from the region, there is evidence from a systematic literature review on policy and prevention approaches for disordered gaming and internet use suggesting that, due to the global prevalence of the phenomena and the geographically dispersed nature of preventative programs, it is imperative that prevention efforts be integrated across national borders while taking cultural differences into consideration [84].

The following recommendations are a recapitulation of the evidence produced by the limited international reviews that have examined the effectiveness of programs and interventions in the prevention of IA [71,84-87].

Early identification and intervention are key to addressing IA among adolescents. Schools can implement evidence-based prevention programs specifically targeting IA. These programs can incorporate educational components, skill-building exercises, and peer support networks to help adolescents develop healthy online habits and balance their time between essential and nonessential activities [87]. Comprehensive school-based programs are essential to effectively address IA. These programs should focus on increasing awareness, develop coping strategies, and promoting responsible digital technology use. These programs can be integrated into the school curricula and should include methods to improve self-control and enhance knowledge of the underlying processes that contribute to excessive online activity, such as constant notifications [88,89]. In addition, schools can implement practical digital well-being initiatives, such as mindfulness exercises and the integration of technology breaks into the daily schedule, to promote healthier digital habits. Moreover, promoting digital literacy and responsible digital technology use is crucial for educating adolescents on the potential risks and consequences associated with excessive digital technology use [90]. Given that adolescents’ behaviors are largely shaped by other external factors such as their families and external environments [21], schools play a crucial role in extending these preventive programs to parents and caregivers. Understanding children’s intentions when interacting with the internet is crucial and emphasizes the need for smarter digital parenting tools that provide enhanced monitoring and guidance. A healthier digital environment can be created by considering intentionality and using smart tools. Devices and programs should be designed to isolate essential and nonessential uses, advising children to avoid installing or visiting purely nonessential social media applications and sites on the same device in which they engage in essential activities.

These recommendations must be supported by designing and enacting policies. Policy makers can design programs targeting the broader environment in which adolescents live, addressing the underlying factors contributing to maladaptive coping, such as reducing academic pressure and improving family support. By reducing dependence on nonessential internet use, these policies, in turn, can empower adolescents to navigate their digital environments more responsibly.

Certain limitations of this study must be considered when interpreting the results. First, due to the cross-sectional design of our study, the generalizability of our findings is limited. Another limitation of our study was that the responses provided by the adolescents were based on self-report, potentially introducing reporting bias. It is possible that the adolescents did not accurately report the time spent on essential and nonessential activities and symptoms of IA. To mitigate the influence of reporting bias, future studies should incorporate objective measures, such as using data on actual screen time, instead of relying solely on self-reported information. Another limitation of this study is that the context of essential and nonessential internet use could have been interpreted by adolescents based on their subjective experiences and perceptions. For example, spending time with friends on social media during the examination period may be considered essential for adolescents who want to receive moral support from their colleagues. Nevertheless, our study left it at the discretion of the participants. The impact of the specific activity or content that adolescents consume or spend excessive time on and the effect it has on them should be scrutinized in more detail and is an area that requires attention. Our sample’s gender imbalance, which was characterized by a higher proportion of female participants, may restrict the generalizability of the results to both genders. Furthermore, we adhered to sample size estimates using the method by Green [49] to guarantee sufficient statistical power for our study. The deliberate choice not to segregate the sample by gender was made to ensure that the sample size remained sufficient for obtaining consistent and dependable regression results. The male sample was relatively too small to allow for that option. Although the existing literature on gender differences in IA is inconclusive, with research presenting inconsistent findings [91-95], it is important to exercise caution when interpreting the results of this study due to the higher proportion of female participants. Future studies should aim for a more even gender distribution to comprehensively investigate any disparities in IA between male and female individuals.

The strength of our study lies in its inclusion of a non-WEIRD population, specifically focusing on adolescent IA in the Middle East, where limited research on this area has been conducted. Another strength of our study is that it addresses the research gap by examining both essential and nonessential internet use in the same sample. While previous research has explored the different online activities that contribute to IA [65,96], few studies have specifically investigated the impact of essential and nonessential internet use within a single study. Another strength of our study lies in the separate analysis of IA factors in early and middle adolescents, which allowed us to capture the unique dynamics and influences shaping IA in each age group, thus enhancing the understanding of IA in relation to developmental stages.

### Conclusions

In conclusion, this study sheds light on the complex relationship between digital technology use and IA among early and middle adolescents. For many years, screen time has been widely used as a prominent measure to assess the extent of IA among adolescents. As the digital society continues to evolve rapidly, relying on screen time as a stand-alone criterion fails to provide a comprehensive understanding of phenomena such as IA. Therefore, it is imperative to distinguish between essential and nonessential digital technology use, acknowledging diverse activities and their implications for a better understanding of individuals’ engagement with digital technology. The findings of our study provide valuable insights for researchers, practitioners, and policy makers in addressing IA among adolescents. By recognizing the distinct nature of internet activities and considering developmental factors, effective interventions and strategies can be developed to promote healthy digital technology use and prevent IA in adolescents.

## Appendix

Multimedia Appendix 1 Survey design.

## Abbreviations

IA: internet addiction
IADQ: Internet Addiction Diagnostic Questionnaire
KMO: Kaiser-Meyer-Olkin
OR: odds ratio
RQ: research question
VIF: variance inflation factor
WEIRD: Western, educated, industrialized, rich, and democratic
