# Supplementary material for: Assessing the Relationship Between the Type of Internet Use and Internet Addiction in Early and Middle Adolescents: Cross-Sectional Study From Qatar
Source: JMIR Hum Factors. 2025 Feb 10;12:e62955. doi: 10.2196/62955 (PMC11851041; doi:10.2196/62955)
Supplement: Multimedia Appendix 1 [file humanfactors_v12i1e62955_app1.docx]

Supplementary files

Survey Design

This supplementary file provides the survey questionnaire in Arabic and English as well as the detailed visualizations of the assumption checks performed for the multiple regression analysis presented in the main manuscript.

**Survey questions in English**

**Excessive Use of Digital Technologies – Research Project**

**Children Survey**

This study is on the “What impact does excessive use of digital technologies have on family cohesion and relationships within the family, health of children and adults, and student learning, and what should parents and policymakers do to address this issue in Qatar.”

You are participating in this study on a voluntary basis. You can withdraw from the study at any point without having to give reasons and with no implications. You have the right to not answer any question if you feel any discomfort.

This instrument is to be completed by adolescents. We assure you that all the information gathered will be anonymous and will only be used for scientific purposes.

1. Age (in years) __________
2. Gender: Male Female
3. Birthplace: Qatar Abroad (specify)_
4. Nationality: Qatari Non-Qatari (specify)___________
5. School name ______________________________
6. School type:

- Public
- Private
- others (specify)____________

1. **Digital Technology Usage**

- How many hours do you use digital technology for **study** purposes **daily** on **weekdays (Sunday – Thursday)**?
- How many hours do you use digital technology for **non-essential reasons** **daily** on **weekdays** **(Sunday – Thursday)**?
- How many hours do you use digital technology for **study** purposes **daily** on **weekends (Friday and Saturday)?**
- How many hours do you use digital technology for **non-essential reasons** **daily** on **weekends (Friday and Saturday)?**

1. Answer the following question about your use of technology for **non-essential reasons**, please:

|  | Yes | No |
| --- | --- | --- |
| Do you feel preoccupied with the Internet (think about previous online activity or anticipate next online session)? |  |  |
| Do you feel the need to use the Internet with increasing amounts of time in order to achieve satisfaction? |  |  |
| Have you repeatedly made unsuccessful efforts to control, cut back, or stop Internet use? |  |  |
| Do you feel restless, moody, depressed, or irritable when attempting to cut down or stop Internet use? |  |  |
| Do you stay online longer than originally intended? |  |  |
| Have you jeopardized or risked the loss of significant relationship, task, educational opportunity because of the Internet? |  |  |
| Have you lied to family members, teachers, or others to conceal the extent of involvement with the Internet? |  |  |
| Do you use the Internet as a way of escaping from problems or of relieving a negative mood (e.g., when feelings helplessness, guilt, anxiety, depression)? |  |  |

1. How do you feel about the amount you **use** digital technology for non-essential reasons?

◯ I am happy with it

◯ I am somewhat happy with it

◯ neither happy nor unhappy with it

◯ I am somewhat unhappy with it

◯ I am unhappy with it

**Survey questions in Arabic**

**الاستخدام المفرط للتكنولوجيا الرقمية – مشروع بحثي**

**استبيان اليافعين**

عزيزي المشارك

ندعوكم للمشاركة في دراسة بحثیة بعنوان "**الاستخدام المفرط للتكنولوجيا الرقمية وتأثيره على الصحة والتعليم والترابط الأسري في قطر"**. أنت تشارك في هذه الدراسة على أساس تطوعي. يمكنك الانسحاب من الدراسة في أي وقت دون الحاجة إلى إبداء الأسباب ودون أي تبعات. لديك الحق في عدم الإجابة على أي سؤال إذا شعرت بعدم ارتياح. هذا الاستبيان مخصص لليافعين من سن 10- 16. ونؤكد لك أننا لن نجمع أي معلومات تدل على إسمك أو شخصيتك كما نؤكد لك أن هذا الاستبيان سيستخدم لأغراض علمية فقط.

1. العمر (بالسنوات): -------
2. الجنس: ذكر أنثى
3. مكان الميلاد: قطر خارج قطر (يرجى ذكر الدولة) ---------
4. الجنسية: قطري غير قطري (يرجى ذكر الجنسية) --------
5. اسم المدرسة:
6. نوع المدرسة:

- حكومي
- خاص
- أخرى (حدد) ----------
- كم ساعة تستخدم التكنولوجيا الرقمية **لأغراض الدراسة** **يوميًا** في **أيام الأسبوع** **(الأحد - الخميس)**؟
- كم ساعة تستخدم التكنولوجيا الرقمية **لأسباب غير ضرورية** **يوميًا** في **أيام الأسبوع (الأحد - الخميس)**؟
- كم ساعة تستخدم التكنولوجيا الرقمية **لأغراض الدراسة** **يوميًا** في **عطلات نهاية الأسبوع (الجمعة والسبت)**؟
- كم ساعة تستخدم التكنولوجيا الرقمية **لأسباب غير ضرورية** **يوميًا** في **عطلات نهاية الأسبوع (الجمعة والسبت)**؟

1. أجب عن السؤال التالي حول استخدامك للتكنولوجيا **لأسباب غير ضرورية**، من فضلك:

|  | نعم | لا |
| --- | --- | --- |
| هل تشعر بأنك مشغول الذهن تجاه ما يجري في الإنترنت (أن تفكّر في نشاط سابق عبر الإنترنت أو تترقب استخدامك المقبل له )؟ |  |  |
| هل تشعر بالحاجة إلى زيادة استخدامك للإنترنت لفترات زمنية أطول من أجل تحقيق الرضا؟ |  |  |
| هل بذلت جهودًا م بشكل متكرر للتحكم في استخدام الإنترنت أو تقليصه أو إيقافه لكنها فشلت ؟ |  |  |
| هل تشعر بالقلق، أو بتقلب المزاج، أو الاكتئاب، أو الانفعال عند محاولتك تقليل أو إيقاف استخدام الإنترنت؟ |  |  |
| هل تبقى متصلاً بالإنترنت لفترة أطول مما كنت تنوي في الأصل؟ |  |  |
| هل عرضت للخطر أو خاطرت بفقدان علاقة مهمة أو فرصة تعليمية بسبب الإنترنت؟ |  |  |
| هل أخفيت الحقيقة على أفراد الأسرة أو المعلمين أو غيرهم لتجنب قول مدى استخدامك للإنترنت؟ |  |  |
| هل تستخدم الإنترنت كطريقة للهروب من المشاكل أو للتخلص من مزاج سلبي (على سبيل المثال، عند الشعور بالعجز، والشعور بالذنب، والقلق، والاكتئاب)؟ |  |  |

1. ما هو شعورك تجاه كمية استخدامك التكنولوجيا الرقمية لأسباب غير ضرورية؟

- أنا سعيد به
- أنا سعيد به إلى حد ما
- لست سعيداً ولا غير سعيد به
- أنا غير سعيد به إلى حد ما
- أنا غير سعيد به

**List of Figures demonstrating assumption checks**

These figures illustrate the diagnostic tests used to validate the underlying assumptions of linearity, normality, and homoscedasticity. The linearity of the sample was checked using residuals vs. predicted plots. Q-Q plots were used to check the normality of the data and residuals vs. dependent plots were used to check for homoscedasticity in the data. The list of figures are as follows:

[Figure 1 : Residual vs Dependent Plot for Multiple Regression Analysis to measure Total Internet addiction in early adolescents (Linearity) 5](#_Toc175316207)

[Figure 2: Residual vs Predicted Plot for Multiple Regression Analysis to measure Total Internet addiction in early adolescents 5](#_Toc175316208)

[Figure 3 QQ Plot for Multiple Regression Analysis to measure Total Internet addiction in early adolescents 6](#_Toc175316209)

[Figure 4: Residual vs Dependent Plot for Multiple Regression Analysis to measure Internal IA symptoms in early adolescents 6](#_Toc175316210)

[Figure 5: Residual vs Predicted Plot for Multiple Regression Analysis to measure Internal IA symptoms in early adolescents 7](#_Toc175316211)

[Figure 6: QQ Plot for Multiple Regression Analysis to measure Internal IA symptoms in early adolescents 7](#_Toc175316212)

[Figure 7: Residuals vs Dependent Plot for Multiple Regression Analysis to measure Total Internet addiction in middle adolescents 8](#_Toc175316213)

[Figure 8: Residuals vs Predicted Plot for Multiple Regression Analysis to measure Total Internet addiction in middle adolescents 8](#_Toc175316214)

[Figure 9: QQ Plot for Multiple Regression Analysis to measure Total Internet addiction in middle adolescents 9](#_Toc175316215)

[Figure 10: Residuals vs Dependent Plot for Multiple Regression Analysis to measure Internal IA symptoms in middle adolescents 10](#_Toc175316216)

[Figure 11: Residuals vs Predicted Plot for Multiple Regression Analysis to measure Internal IA symptoms in middle adolescents 10](#_Toc175316217)

[Figure 12: QQ Plot for Multiple Regression Analysis to measure Internal IA symptoms in middle adolescents 11](#_Toc175316218)

# Early Adolescents

## Total Internet Addiction


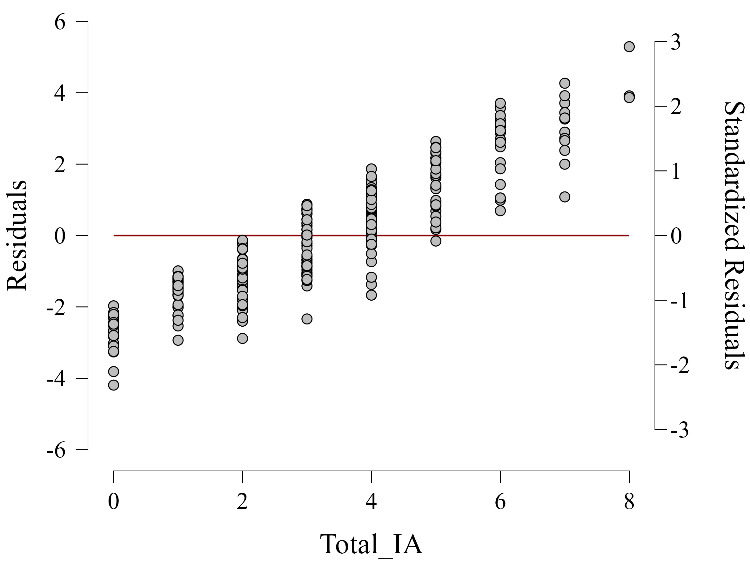


Figure 1 : Residual vs Dependent Plot for Multiple Regression Analysis to measure Total Internet addiction in early adolescents (Linearity)


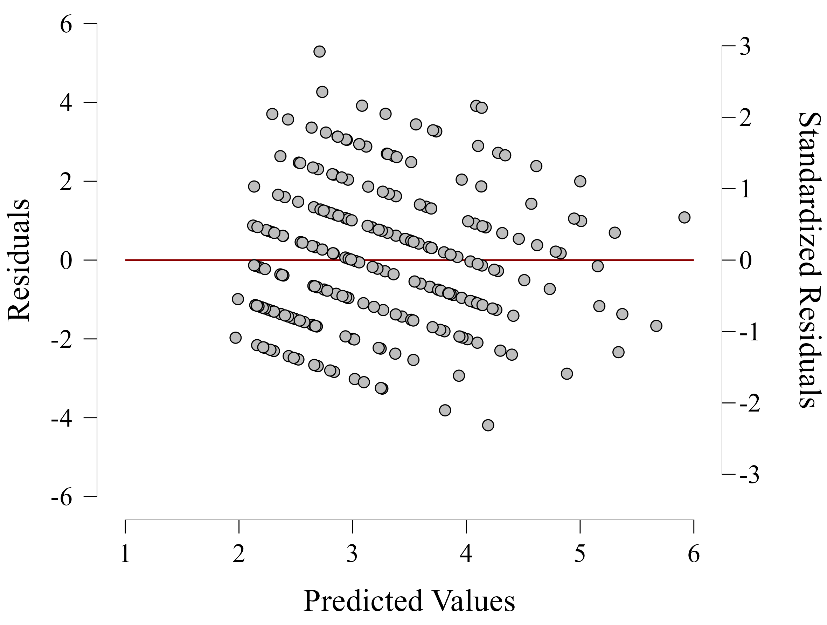


Figure 2: Residual vs Predicted Plot for Multiple Regression Analysis to measure Total Internet addiction in early adolescents


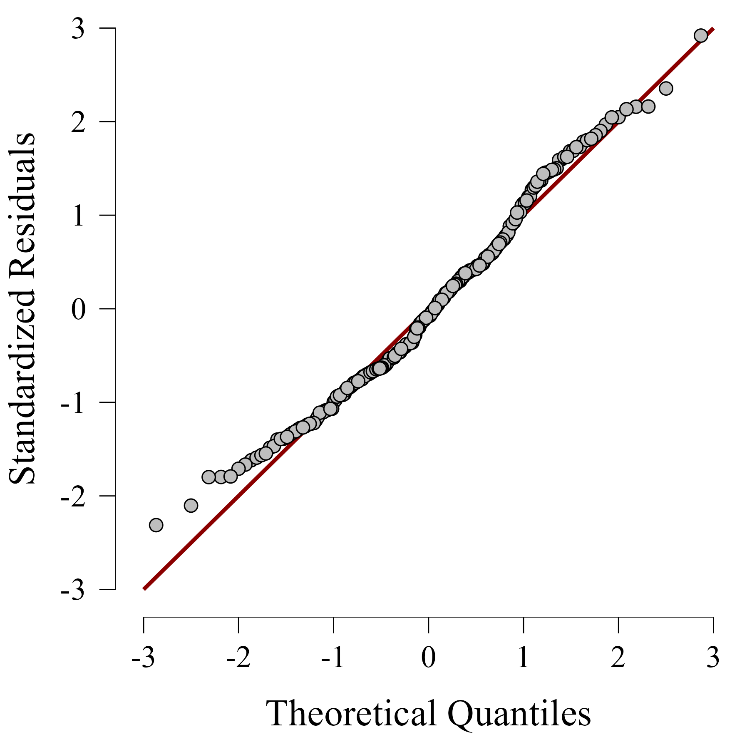


Figure 3 QQ Plot for Multiple Regression Analysis to measure Total Internet addiction in early adolescents

# Early Adolescents

## Internal IA Symptoms


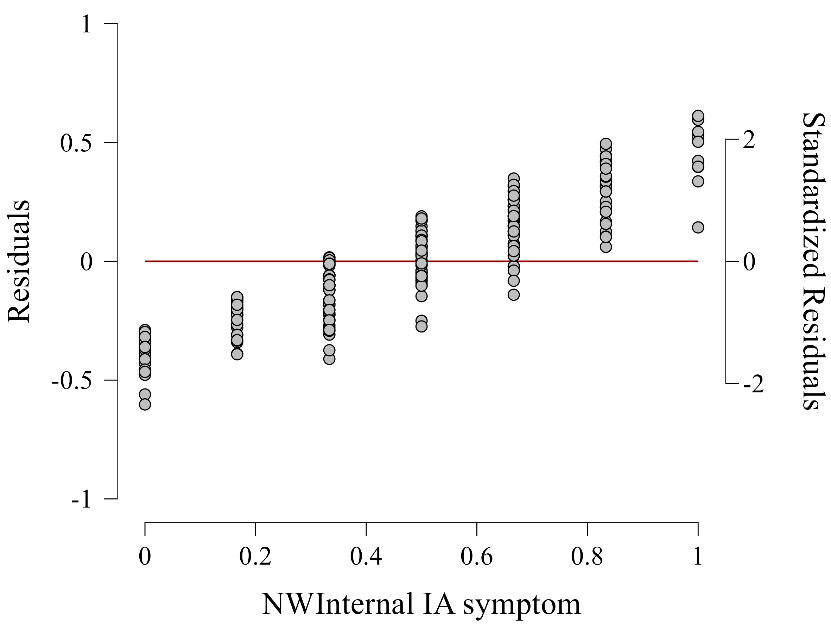


Figure 4: Residual vs Dependent Plot for Multiple Regression Analysis to measure Internal IA symptoms in early adolescents


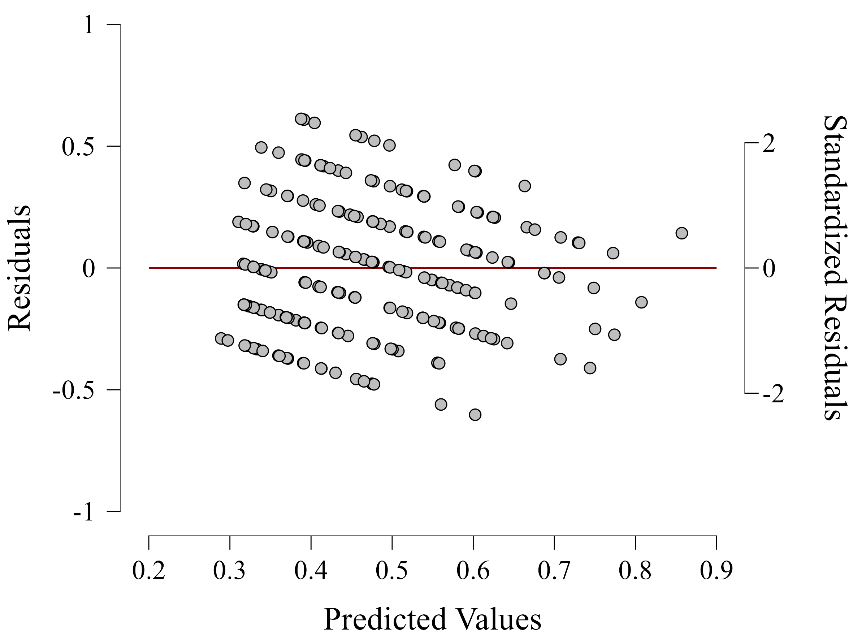


Figure 5: Residual vs Predicted Plot for Multiple Regression Analysis to measure Internal IA symptoms in early adolescents


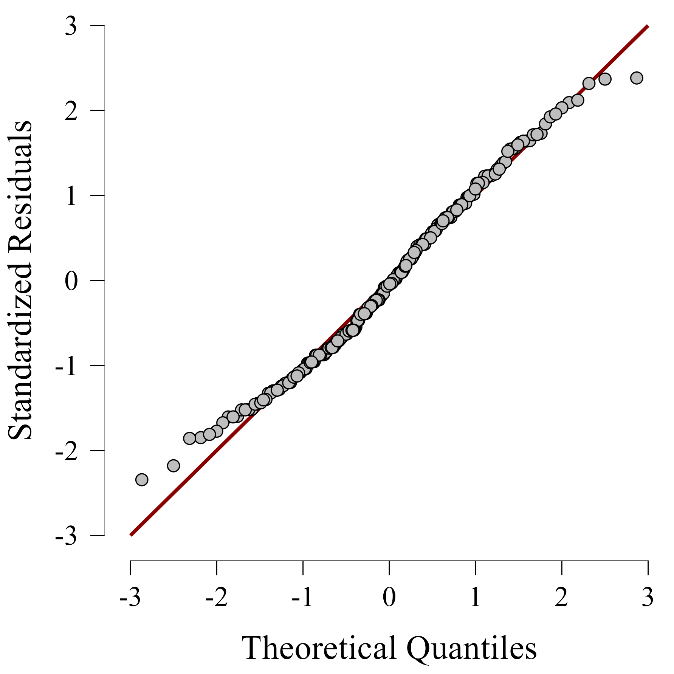


Figure 6: QQ Plot for Multiple Regression Analysis to measure Internal IA symptoms in early adolescents

# Middle Adolescents

## Assumption checks to measure Total Internet Addiction


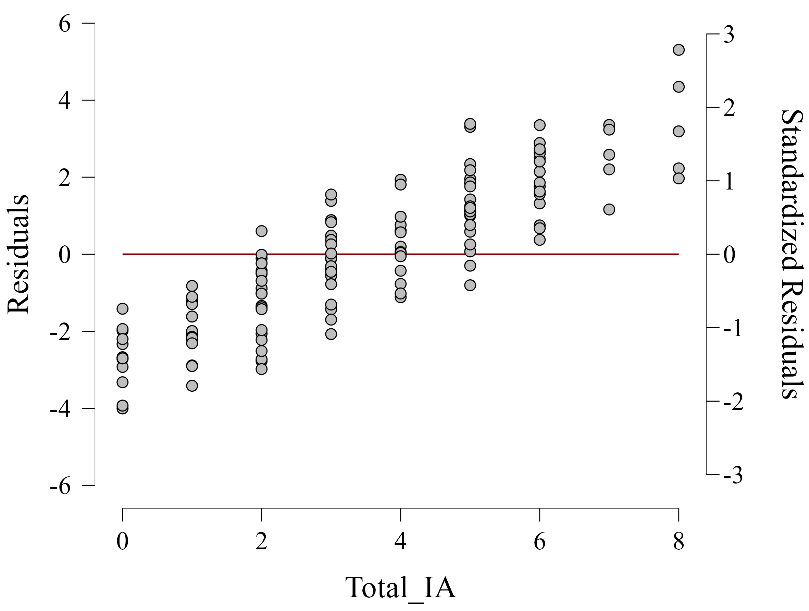


Figure 7: Residuals vs Dependent Plot for Multiple Regression Analysis to measure Total Internet addiction in middle adolescents


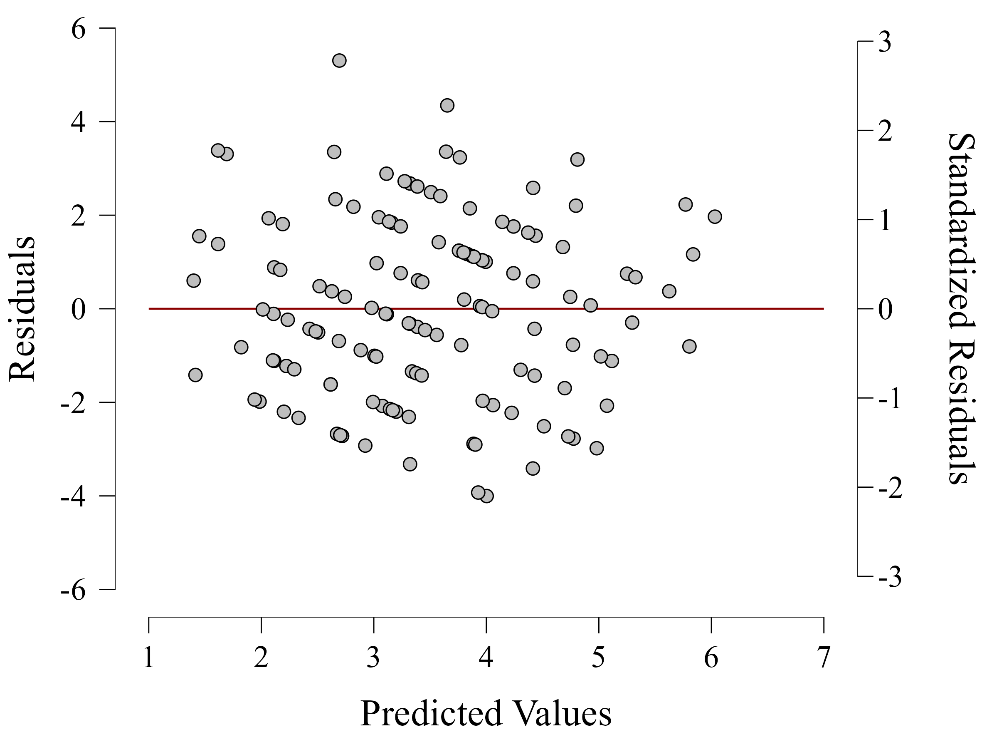


Figure 8: Residuals vs Predicted Plot for Multiple Regression Analysis to measure Total Internet addiction in middle adolescents


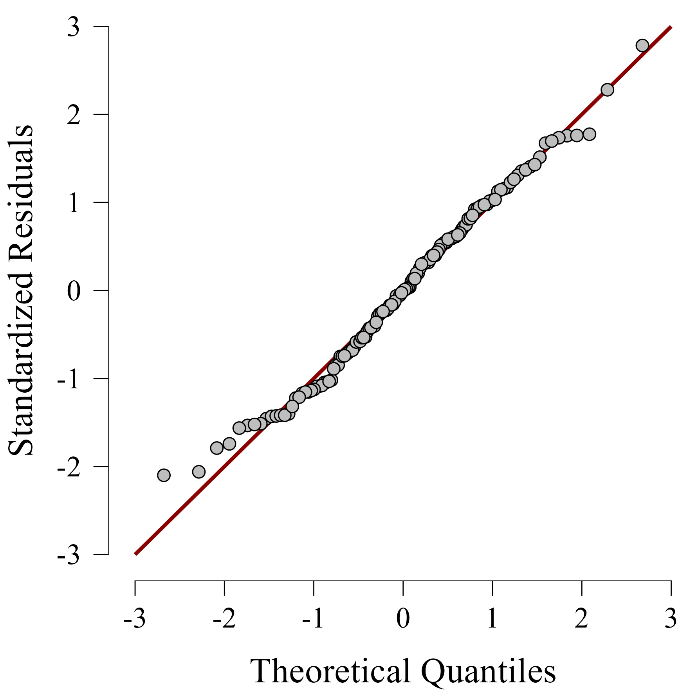


Figure 9: QQ Plot for Multiple Regression Analysis to measure Total Internet addiction in middle adolescents

# Middle Adolescents

## Assumption checks to measure Internal IA symptoms


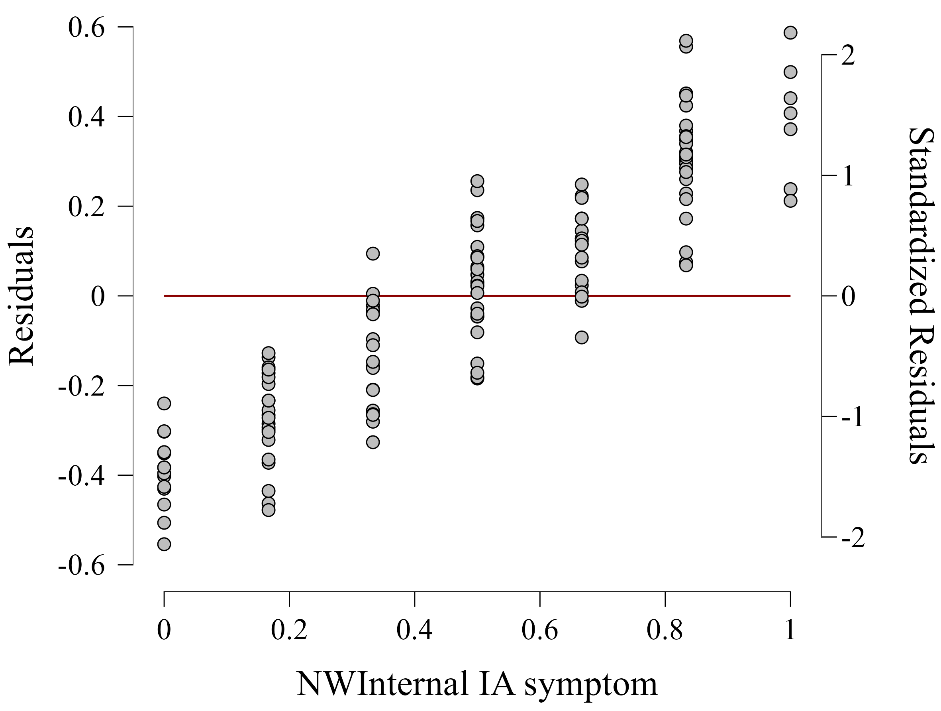


Figure 10: Residuals vs Dependent Plot for Multiple Regression Analysis to measure Internal IA symptoms in middle adolescents


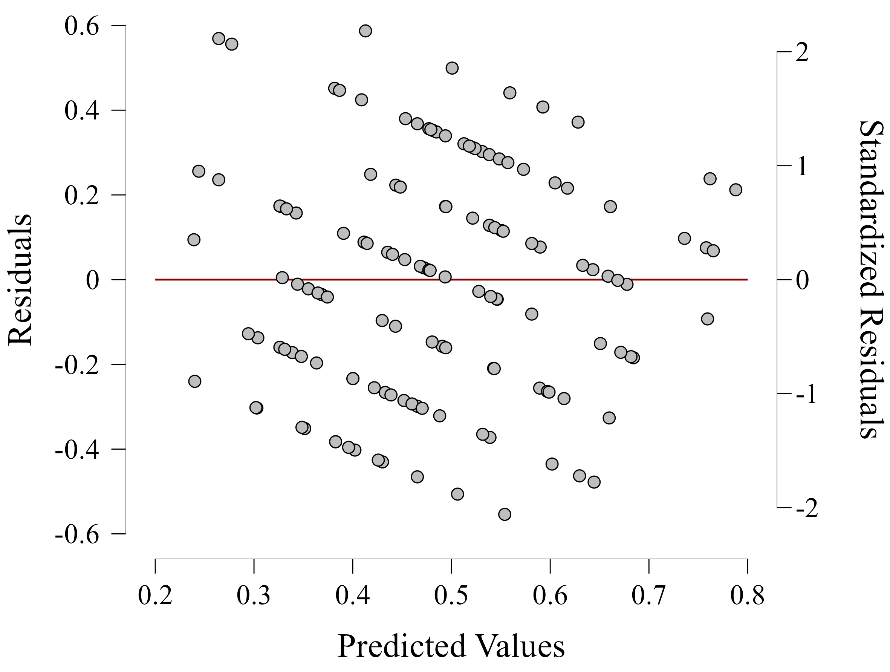


Figure 11: Residuals vs Predicted Plot for Multiple Regression Analysis to measure Internal IA symptoms in middle adolescents


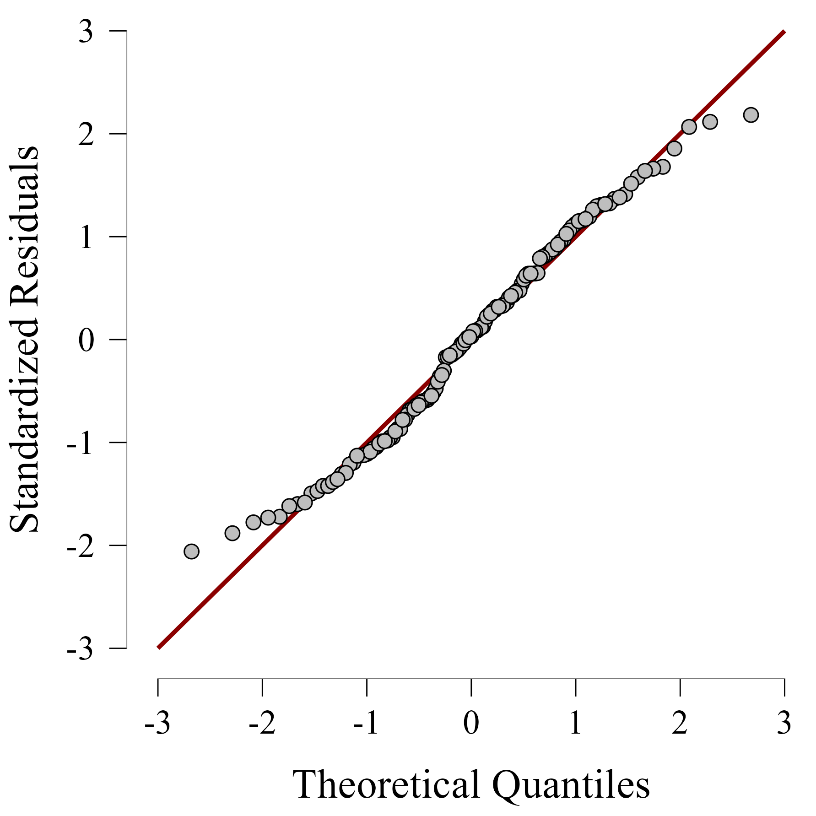


Figure 12: QQ Plot for Multiple Regression Analysis to measure Internal IA symptoms in middle adolescents
